# Approaching humans for food is a behavior transmitted from mothers to offspring in a large herbivore

**DOI:** 10.1093/beheco/arag034

**Published:** 2026-04-06

**Authors:** Jane Faull, Laura L Griffin, Cong Yu, Bawan Amin, Adam F Smith, Amy Haigh, Anna David, Sarah Redmond, Niamh Collins, Simone Ciuti

**Affiliations:** Laboratory of Wildlife Ecology and Behaviour, UCD School of Biology and Environmental Science, Room E3.37, Science Centre East, University College Dublin, Dublin 4, Ireland; Laboratory of Wildlife Ecology and Behaviour, UCD School of Biology and Environmental Science, Room E3.37, Science Centre East, University College Dublin, Dublin 4, Ireland; Department of Forest Resources Management, University of British Columbia, 2424 Main Mall, Vancouver, BC, Canada V6T 1Z4; Laboratory of Wildlife Ecology and Behaviour, UCD School of Biology and Environmental Science, Room E3.37, Science Centre East, University College Dublin, Dublin 4, Ireland; Laboratory of Wildlife Ecology and Behaviour, UCD School of Biology and Environmental Science, Room E3.37, Science Centre East, University College Dublin, Dublin 4, Ireland; The Frankfurt Zoological Society, Bernhard-Grzimek-Allee, Frankfurt, Hesse, Germany; Department of Wildlife Ecology and Management, Faculty of Environment and Natural Resources, University of Freiburg, Freiburg, Baden-Württemberg, Germany; Laboratory of Wildlife Ecology and Behaviour, UCD School of Biology and Environmental Science, Room E3.37, Science Centre East, University College Dublin, Dublin 4, Ireland; Laboratory of Wildlife Ecology and Behaviour, UCD School of Biology and Environmental Science, Room E3.37, Science Centre East, University College Dublin, Dublin 4, Ireland; Laboratory of Wildlife Ecology and Behaviour, UCD School of Biology and Environmental Science, Room E3.37, Science Centre East, University College Dublin, Dublin 4, Ireland; Laboratory of Wildlife Ecology and Behaviour, UCD School of Biology and Environmental Science, Room E3.37, Science Centre East, University College Dublin, Dublin 4, Ireland; Laboratory of Wildlife Ecology and Behaviour, UCD School of Biology and Environmental Science, Room E3.37, Science Centre East, University College Dublin, Dublin 4, Ireland

**Keywords:** artificial feeding, human-wildlife coexistence, maternal behavior, human-wildlife feeding interactions, cultural transmission, neonatal personality

## Abstract

Human-wildlife interactions, particularly artificial feeding, have led to significant behavioral changes in wildlife. Begging for food is a behavior commonly seen in species like gulls, seals and foxes across social media. There are concerns about whether this behavior is passed from parent to offspring, either through genetic inheritance or cultural transmission. In our model peri-urban deer population in Dublin, Ireland, we observed a subset of individuals consistently begging for food. We conducted a 6-year longitudinal study involving 255 fawns from 146 mothers to investigate the transmission of begging behavior. We found that offspring of consistently begging mothers were more likely to beg for food than those whose mothers avoided humans. Neonatal personality also played a role in shaping offspring behavior: bolder (more risk taking) fawns were more likely to beg. Our novel results provide empirical evidence of intergenerational transmission of begging behavior. Combined with prior findings that begging mothers have heavier fawns at birth that grow faster, and have higher first-year survival rates, our study suggests this behavioral trait may become more prevalent over generations, aligning with increasing reports of human-wildlife feeding interactions in urban areas.

## Teaser

Human wildlife interactions like feeding wildlife can influence their behavior. Over 6 years, we found mothers who begged for food were more likely to have fawns that adopt the same behavior. Bolder fawns were also more likely to beg, demonstrating that both mother’s behavior and fawn personality shape offspring begging behavior. Since begging leads to heavier, faster-growing fawns, this behavior might become more common over generations, reflecting rising human-wildlife feeding interactions in peri-urban and urban areas.

## Introduction

Human-wildlife interactions have shaped the behavior of wildlife for generations (Goumas et al. 2020). Activities like hunting and fishing are well known for promoting artificial selection by favoring specific behavioral and morphological traits in animals (Darimont et al. 2009; Ciuti et al. 2012). Evolutionary changes have been suggested in Stone's sheep (*Ovis dalli stonei*), with selective hunting favoring individuals with larger horns, where marked morphological changes have been observed both in horn shape and size, contrasting sexually selected patterns (Douhard et al. 2016). Preferentially harvesting fast growing, larger individuals in fisheries has led to physiological, developmental, morphological, behavioral, and life history traits to evolve including declines in egg volume, larval size, and fecundity (Walsh et al. 2006).

More recently, however, it has been discovered that less consumptive interactions such as humans feeding wildlife can artificially select for certain traits by rewarding individuals that interact with humans with additional (normally high calorie) food (Griffin et al. 2022; Faull et al. 2024). This could have considerable impacts on population structures over time with more and more individuals participating in feeding interactions and wildlife begging humans for food. Yet little is understood about how these behaviors are passed between individuals within a population and particularly, whether they are transmitted from parent to offspring.

The ontogeny of behavioral traits, such as begging for food, is shaped by a combination of genetic, environmental, and social mechanisms that interact throughout development. Genetic predispositions may influence the likelihood of certain behavioral expressions, while environmental conditions—both abiotic (eg, temperature; van Baaren et al. 2005) and biotic (eg, predator presence; Abrams and Matsuda 1993)—can modify how these behaviors are expressed or reinforced. Social learning further contributes to behavioral variation, as individuals acquire behaviors from conspecifics through horizontal transmission among peers or vertical transmission across generations (Dugatkin 2014). Such transmission allows behaviors that enhance survival or reproductive success to spread within populations (Manassa et al. 2014). Parental influences may also shape behavioral development, as shown in Egyptian fruit bats (*Rousettus aegyptiacus*), where urban-adapted mothers produce offspring that exhibit similar boldness and faster learning compared with rural counterparts (Harten et al. 2021). These findings highlight the importance of parental and early-life effects as key mechanisms linking individual developmental processes with population-level behavioral variation.

Indeed maternal effects can influence offspring behavior, whereby the maternal phenotype or genotype influences the phenotype of the offspring in a causal way (Wolf and Wade 2009). Similarly maternal environment and care in early life can impact the physical and social development of offspring, which can in turn carry forward to the next generation. For example, in rodents a grandmother's impact on her granddaughter's behavior can be mediated through the care she provided to the granddaughter's mother (Champagne 2008). This intergenerational shaping of behavior demonstrates how inherited, learned, and socially transmitted traits can accumulate over time within populations.

There are several examples of offspring behaviors being impacted by their parents, either by being directly passed down or heavily influenced by the parent (Francis et al. 1999; Reddon 2012; Amin et al. 2021; Vanden Broecke et al. 2021). Schuett et al. (2013) showed that zebra finches (*Taeniopygia guttata*) adopted the exploratory tendencies of their foster parents over their biological parents illustrating nongenetic transmission of behaviors.

In addition to the well-established biological and social drivers described above, human activities and urbanization are shaping wildlife behavior. By providing novel food sources and rewarding bolder (more risk taking) individuals, humans may inadvertently create new ecological niches that differ across behavioral types (Miranda et al. 2013; Baxter-Gilbert et al. 2019; Griffin et al. 2022). Bolder individuals, for example, may adjust their behavior to exploit these opportunities by approaching people or soliciting food directly from them (Dasgupta et al. 2025), while shyer conspecifics remain reliant on natural foraging strategies. Repeated reinforcement of successful behavior adjustments can promote their persistence and social transmission within populations. Over longer timescales, if bold individuals experience higher survival or reproductive success, these behavioral tendencies could also contribute to changes in the behavioral composition of the population. Bolder (more risk taking) individuals are fed and rewarded in many different environments and contexts, with wildlife feeding having steadily gained popularity across the globe (for a review see Griffin and Ciuti 2023). For example, it is common to feed garden birds, supplementing their diet in the colder winter months and providing people living in urban areas with an opportunity to interact with nature (Cox and Gaston 2016). This practice has become so widespread and popular that there is now a multibillion-dollar industry dedicated to it (Plummer et al. 2019). While wildlife feeding is often associated with, and isolated to, animals living in urban and peri-urban settings, Shutt et al. (2021) have demonstrated through blue tit (*Cyanistes caeruleus*) fecal metabarcoding that its impacts are more widespread and suggest that it is likely that all blue tits in the UK have access to supplementary human food. Essentially this means that every blue tit in the UK could have access to human provided foods (Shutt et al. 2021). Feeding wildlife also attracts tourists, as noted across multiple species from barbary macaques (*Macaca sylvanus*) in the Middle Atlas Mountains, Morocco to bottlenose dolphins (*Tursiops sp.*) in Shark Bay, Australia (Mann et al. 2000; Borg et al. 2014). Increased feeding by tourists can cause behavioral changes in wildlife populations. For example, Donaldson et al. (2012) have shown that increased time spent in areas with high feeding opportunities and around other individuals that accepted food from humans resulted in increased food acceptance behaviors in wild dolphins.

Further expanding on this using a peri-urban population of fallow deer (*Dama dama*) in the Phoenix Park in Dublin, Ireland, we aimed to investigate whether the likelihood of offspring to engage with people feeding is related to their mother's tendency to take food from humans. Despite discouragement from park management and campaigns aimed to deter visitors from feeding the deer (Griffin et al. 2023a, 2023b), feeding has only gained popularity in recent years (Griffin and Ciuti 2023). Griffin et al. (2022) has identified distinct behavioral traits in the population of deer in Phoenix Park with some individuals actively approaching and engaging with humans attempting to feed (known as consistent/occasional beggars) and others evading human interactions (rare beggars, ie, those that are present in the herd, but generally avoid direct interactions [Griffin et al. 2022]). There is no paternal care in this species (Chapman et al. 1975), which is notably sexually segregated outside the mating season (Ciuti and Apollonio 2008). Once a fawn is born and has joined the female herd, their only adult behavioral influences for the first few months come from their mothers and other females in the population. As large herds gather in feeding hotspots in the Phoenix Park and fallow deer fawns spend most of their early life with their mothers (often in these large herds) we question what impact this association is having on their behaviors.

We hypothesize that fawn begging behavior in the peri-urban population of Phoenix Park is associated with maternal begging tendencies, such that offspring of frequent beggars are more likely to beg as they grow. While our study cannot disentangle whether this reflects additive genetic effects, maternal influences, or social learning, establishing such an intergenerational link would provide novel evidence for the potential transmission of begging behavior in large herbivores. This would also provide key parameters (eg, estimated heritability of begging behavior) for future simulations predicting how the proportion of begging individuals would change in this population over time. Currently it stands that 1 quarter of the deer actively approach and beg humans for food (Griffin et al. 2022). Our main hypothesis can be further broken down into 2 key questions:

1. Is there additive genetic variation underlying begging behavior in fawns, such that related individuals (eg, mothers–offspring, siblings, cousins) resemble one another in their tendency to beg?
2. Assuming that begging behavior is a repeatable trait in mothers (Griffin et al. 2022), is this behavior in fawns regulated by their tendency to take more risky behaviors and therefore approach humans? More specifically to our study case, can begging behavior be predicted by a fawn's neonatal personality? Therefore, are bold neonate fawns more likely to become beggars later in life?

## Methods

### Study site and population

We conducted our study in the Phoenix Park in Dublin City center, Ireland (53.3559°N, 6.3298°W), an enclosed urban park spanning 7 km^2^ that is home to a population of ∼600 fallow deer. Adult deer face no natural predation issues, but neonates can be preyed upon by foxes (*Vulpes vulpes*) and unleashed dogs (Conteddu et al. 2023). Population levels are managed by the Office of Public Works (Irish government agency responsible for the management of deer in the park) through annual culls. The population is free ranging and regularly encounters the public due to an estimated 10 million annual visitors (OPW, official data).

### Fawn capture protocol

The fawning season begins annually in early June when pregnant females isolate themselves from the herd to birth their fawns in bedsites (Amin et al. 2021; Faull et al. 2024). During the first weeks of life most of the newborn fawns in the population were captured and received uniquely identifiable ear tags to be studied longitudinally. Each tag has a unique color, number, or letter number combination to help them remain recognizable in the field as they mature and join the herd. Captures took <15 min and are carried out under the supervision of a certified wildlife biologist and approved under the animal care permit UCD-AREC-E-18-28, which also covers all noninvasive behavioral observations described here. Upon discovery, fawns were promptly caught using circular fishing nets (1 to 1.5 m diameter) with elongated handles (1 to 1.5 m length). The fawn's alertness prior to capture was scored from 0 (inactive) to 1 (active, head up, ears up, attempt to escape), following Amin et al. (2021). This measurement is later represented as meancap in the modeling stages.

In accordance with the well-established protocol introduced by Amin et al. (2021), we conducted a standardized behavioral assay on the immobilized fawn commonly within the first 2 to 3 weeks after birth. This assay collected 1 physiological (heart rate after handling and prior to release) and 1 behavioral (latency to leave upon release) trait expression of the innate reaction of neonate fawns to human handling. Both traits have been shown to be highly repeatable over multiple captures and recaptures within the same fawning season, with yearly repeatability estimates ranging from 0.25 to 0.39 (see Amin et al. 2022a, 2022b), deemed as correlated neonatal personality traits and linked to early-life history traits (Amin et al. 2022a). Heart rate (physiological trait) was measured using a Lightweight Dual Head Stethoscope (MDF), firstly immediately upon capture (baseline physiological state) and secondly before release, the latter representing the physiological reaction to human handling (trait 1). Fawns were then placed in a bag for weighing and the bag was unzipped facing away from the capture team and opened to allow them to escape. The time it took for a fawn to react (stand up or walk/run away) was recorded in seconds using a stopwatch and termed latency to leave—ie, our behavioral trait 2 (Amin et al. 2021), representing the behavioral reaction to human handling. Note that latency was later converted into a categorical binomial during data analysis as outlined in the model description, basically distinguishing fawns immediately moving away from the bag (leavers) opposed to those who stayed (see below for full details).

In addition to these focal traits, fawns were weighed in the 100L cloth bag using a digital scale (resolution: 0.01 kg—Dario Markenartikelvertrieb). Visibility of the site to predators (ie canids) was recorded by measuring how visible a shape of the size of a standing fawn was at the height of a fox (the most common natural predator of fawns in this site) in the 4 cardinal directions from a distance of 10 m (Amin et al. 2022a, 2022b). We also recorded weather conditions, vegetation cover and air temperature (recorded using a digital thermometer).

### Determining mother-fawn relationships

Due to a lack of pedigree data on the identity of fathers and no paternal care (Chapman et al. 1975), we focused on the maternal connections within the population as they could be determined through behavioral observations (Griffin et al. 2023a, 2023b; Faull et al. 2024). Once fawns had matured to the point of joining the female herd (starting from early July after the birth season being centered in June), they were monitored for interactions with adult females, who were then classified as mothers. To confirm a mother-offspring relationship we required at least 2 independent observations (ie not conducted on the same observation session or day) of following, true suckling or social grooming behavior between mother and fawn (Griffin et al. 2023a, 2023b). Following behavior was recorded when the fawn's behavior was observed to closely mirror its mother, staying right behind her, moving when she moved, and stopping when she stopped. We use the term “true suckling” rather than just suckling as it is common for fawns to try and feed from females that are not their mothers, known as allosuckling (Roulin 2002). True suckling occurred when the mother was fully aware of the fawn's presence (usually having been approached from the front) and allowed it to suckle without protest. Allosuckling and true suckling can be clearly differentiated when multiple fawns try to take advantage of a suckling opportunity by trying to feed from a mother who has allowed her own fawn to suckle (Pelabon et al. 1998). Mothers respond aggressively, driving away the other fawns and only allowing her own to suckle. Social grooming both from doe to fawn and fawn to doe were recorded. While these interactions can occasionally take place between nonrelated females and fawns, females would not tolerate them on multiple independent occasions with a fawn that is not their own. From these interactions we were able to build a pedigree that includes both male and female offspring and maternal/grandmother lineage, notedly it does not include paternal/grandfather relationships due to lack of paternal care in the species and lack of genetic data available to us.

### Willingness to accept food from humans (ie begging behavior)

Observational studies were conducted to record any human-deer interactions and, due to their unique ear tags, we were able to recognize and score individuals along a behavioral continuum that classified their tendency to avoid or approach humans and participate in feeding interactions (Griffin et al. 2022, Griffin et al. 2023a, 2023b; Faull et al. 2024). Important stages in the female biological cycle occur during spring-summer (late gestation, birth, and early weaning [Ciuti et al. 2006]) so we conducted our observations from May to July each year (2018 to 2024). The park was subdivided and sampled on a scheduled basis to avoid temporal or spatial bias. Starting from dawn to dusk and using a stratified sampling design that accounted for factors like day of the week, time of day and area sampled, we observed both male and female herds within the park (Griffin et al. 2022).

This species sexually segregates (Ciuti and Apollonio 2008) with female offspring remaining with their mothers into adulthood and male offspring spending a variable amount of their early lives with their mothers before moving to the male herd (from a few months to 1 to 2 years of age) (Yu et al, in revision). It should be noted that most males are no longer spotted with their mothers after 1 to 1.5 yrs of age. In terms of female offspring, who potentially remain with their mothers in the female herd, they experience weakening bonds with their mother in each successive year of our 6 year study as she produces more offspring and focuses her attention on her newer fawns.

Observations began when a herd had been located and if multiple herds were present in a sector, one was selected at random. The herd size and individuals present were recorded as well as the presence or absence of humans available to feed within 250 m in all directions. Any interactions with humans were recorded whether the deer actively participated or not and we noted the number of people involved, time of day, duration of interaction and any food accepted. We also noted any additional non feeding interactions like petting, harassing, and dominance displays. See Griffin et al. (2022) for the outline of the full collection protocol.

### Data handling and statistical analysis aimed at testing our a priori hypotheses

We combined data from fawns born within the study timeline with feeding data from the entire population to form our final datasets. Mother ID could appear across multiple rows if she had multiple fawns across the study period (2018 to 2024). We then fit 2 models using Bayesian mixed models based on Markov Chain Monte Carlo (MCMC) approximations from the *MCMCglmm* package (Hadfield 2010) in R 4.3.2 (R Core Team 2023) to test our 2 a priori hypotheses described at the end of the introduction (H1: there could be additive genetic variation underlying begging behavior in fawns, such that related individuals (eg, mothers–offspring, siblings, cousins) resemble one another in their tendency to beg, and H2: assuming begging behavior is a repeatable trait in mothers, begging behavior in fawns could be predicted by a repeatable trait ie bold neonatal personality). Our first model estimates the amount of additive genetic variation (which can include some nongenetic components given the data) for the begging trait by comparing the phenotypes of mothers and their offspring (both male and female). Not only does it compare individuals across generations, but it leverages the full pedigree structure to compare phenotypes among individuals within the same generation including cousins and siblings for example (Kruuk 2004; Wilson et al. 2010). Therefore, hypothesis 1 was tested using a univariate mixed model with the following structure:

**1. Is there additive genetic variation underlying begging behavior in fawns, such that related individuals (eg, mothers–offspring, siblings, cousins) resemble one another in their tendency to beg??**

(*N* = 11,986 observations of human-deer interactions)


$$\begin{array}{rl} \mathrm{Begging} & =\;\beta_{0}\;+\;\beta_{1}\times \mathrm{Herd}\;\mathrm{Siz}e_{i}\;+\;\beta_{2}\times \mathrm{Herd}\;\mathrm{Siz}e_{i}^{2}\;+\;\beta_{3}\times \mathrm{Herd}\;\mathrm{Se}x_{i}\\ & \;+\;\beta_{4}\times \mathrm{Mont}h_{i}\;+\;\beta_{5}\times \mathrm{Yea}r_{i}\;+\;\beta_{6}\times \mathrm{Day}\;\mathrm{of}\;\mathrm{Wee}k_{i}\;+\;\beta_{7}\\ & \;\times \mathrm{Monitoring}\;\mathrm{Tim}e_{i}\;+\;\beta_{8}\times (\mathrm{Monitoring}\;\mathrm{Tim}e_{i}{)}^{2}\;+\;\beta_{9}\\ & \;\times \sqrt{\mathrm{Total}\;\mathrm{Peopl}e_{i}}+\;\beta_{10}\times {\left(\sqrt{\mathrm{Total}\;\mathrm{Peopl}e_{i}}\right)}^{2}+\;\beta_{11}\\ & \;\times \;\mathrm{Time}\;\mathrm{of}\;\mathrm{Da}y_{i}\;+\;\beta_{12}\times \;\mathrm{Time}\;\mathrm{of}\;\mathrm{Da}y_{i}^{2}\;+\;\beta_{13}\times \mathrm{Ag}e_{i}\;+\;\beta_{14}\\ & \;\times \mathrm{Ag}e_{i}^{2}+\;u_{i}\;+v_{j}+w_{k}+t_{l} \end{array}$$

Where the 4 random effects are specified as:

$u_{i}∼N(0,\sigma_{\mathrm{animal}}^{2})$ which represents additive genetic effect (pedigree with data on the mother-fawn pairings).

$v_{j}∼N(0,\sigma_{\mathrm{ID}}^{2})$ which represents individual identity (repeated measurement of the same individual deer).

$w_{k}∼N(0,\sigma_{\mathrm{group}}^{2})$ which represents group identity random effect (which allows the model to take into account observations of multiple individual deer within the same group).

$t_{l}∼N(0,\sigma_{\mathrm{year}}^{2})$ which represents year-level random effect.

This approach models begging behavior based on willingness to interact with humans and feeding interactions (response variable: to beg or not to beg during a given behavioral observation) while considering important confounding factors like herd size (included because larger herds attract more attention and are fed more often (Griffin et al. 2022; Faull et al. 2024), the predominant sex of the herd (categorical, sex defined based on more than 50% males or females and included as a confounding variable because male groups have a greater tendency to beg [Griffin et al. 2022]), month of study (fitted as categorical predictors to account for differences in monthly conditions), day of the week (dow, 3 levels: Friday, Saturday, Sunday), duration of time spent monitoring the herd (monitoring time to account for sampling effort), number of people present (representing the total number of people that attempted to interact with the herd during the observation session), time of day (average time of entire feeding observation for that group), and age of the individuals (see Griffin et al. 2022). Note that the high sample size of our observational database derives from the fact that during each herd observation we collected data on the identity of deer who did not beg for food, as well as all the instances in which ear-tagged deer did beg for food approaching park visitors. All numerical predictors were scaled (row value minus the sample mean eventually divided by the sample standard deviation) to improve model convergence and their quadratic included where appropriate to account for nonlinearity in the data. We ran the Monte Carlo Markov chains for 3,500,000 iterations with a burn-in interval of 50,000 and a thinning interval of 1000. A total of 3450 iterations were sampled to estimate parameters for this model. Since begging behavior is a categorical binomial variable, we constrained its prior variance of the fixed effects to 1 (Hadfield 2010), and we specified a weakly informative prior variance (*V*) and degrees of freedom (*n*) for random effects (*V* = 1, *n* = 0.002).

Repeatability of begging behavior was estimated as the proportion of total variance attributable to individual identity (additive genetic plus observation-level variance), accounting for residual variance fixed at *π*^2^/3 under a threshold model. Heritability of begging behavior was estimated from the *MCMCglmm* model's variance components as the proportion of additive genetic variance (animal random effect) relative to total phenotypic variance, which included the variances of all random effects and the fixed residual variance of π^2^⁄3, as appropriate for a binary trait analysed under a threshold model framework (Hadfield 2010). Posterior means and 95% credible intervals were obtained from the MCMC samples.

To test our second hypothesis, we fit the following multivariate mixed model:

**2. Can begging behavior be predicted by a fawn's neonatal personality? (*N* = 11,282 observations)**
 
  $$\begin{array}{rl} \mathrm{Heart}\;\mathrm{rate} & =\;\beta_{0}\;+\;\beta_{1}\times \mathrm{AirTem}p_{i}\;+\;\;\beta_{2}\times (\mathrm{AirTem}p_{i}{)}^{2}\\ & \;+\;\beta_{3}\times \mathrm{Hou}r_{i}^{2}\;+\;\beta_{4}\times \mathrm{MeanCa}p_{i}\\ & \;+\;\beta_{5}\times \mathrm{Weigh}t_{i}\;+\;\beta_{6}\times {\mathrm{Year}}_{i}+\;\beta_{7}\times \mathrm{Se}x_{i}\;+\;u_{i}\;+v_{j} \end{array}$$
   
  $$\begin{array}{rl} \mathrm{Latency} & =\;\beta_{0}\;+\;\beta_{1}\times \mathrm{Hou}r_{i}\;+\;\;\beta_{2}\times \mathrm{Captur}e_{i}\;+\;\beta_{3}\times \mathrm{MeanCa}p_{i}\\ & \;+\;\beta_{4}\times \mathrm{Se}x_{i}+\beta_{5}\times \mathrm{Visibilit}y_{i}\;+\;\beta_{6}\times \mathrm{Weigh}t_{i}\\ & \;+\beta_{7}\times \mathrm{Yea}r_{i}+u_{i}\;+v_{j} \end{array}$$
  $$\begin{array}{rl} \mathrm{Begging}\;= & \beta_{0}\;+\;\beta_{1}\times \mathrm{Herd}\;\mathrm{Siz}e_{i}\;+\;\beta_{2}\times \mathrm{Herd}\;\mathrm{Siz}e_{i}^{2}\\ & +\;\beta_{3}\times \mathrm{Herd}\;\mathrm{Se}x_{i}\;+\;\beta_{4}\times \mathrm{Mont}h_{i}\;+\;\beta_{5}\times \mathrm{Yea}r_{i}\;+\;\beta_{6}\\ & \times \mathrm{Day}\;\mathrm{of}\;\mathrm{Wee}k_{i}\;+\;\beta_{7}\times \mathrm{Monitoring}\;\mathrm{Tim}e_{i}+\;\beta_{8}\\ & \times (\mathrm{Monitoring}\;\mathrm{Tim}e_{i}{)}^{2}\;+\;\beta_{9}\times \sqrt{\mathrm{Total}\;\mathrm{Peopl}e_{i}}+\;\beta_{10}\\ & \times {\left(\sqrt{\mathrm{Total}\;\mathrm{Peopl}e_{i}}\right)}^{2}+\;\beta_{11}\times \;\mathrm{Time}\;\mathrm{of}\;\mathrm{Da}y_{i}\;+\;\beta_{12}\\ & \times \;\mathrm{Time}\;\mathrm{of}\;\mathrm{Da}y_{i}^{2}\;+\;\beta_{13}\mathrm{Ag}e_{i}\;+\;\beta_{14}\times \mathrm{Ag}e_{i}^{2}+\;u_{i}\;+v_{j} \end{array}$$

Random effects were included at 2 levels:


$$u_{i}^{\left(t\right)}∼\;N(0,\;G^{\left(t\right)})$$

Representing the effect of individual identity (eartag), with an unstructured covariance matrix across traits.


$$v_{j}^{\left(t\right)}∼\;N(0,\;R^{\left(t\right)})$$

Representing the group identity random effect, with trait-specific residual covariance structures.

Heart rate (log transformed), latency and begging behavior were the 3 response variables with a different set of predictors as listed above and fully described below. The predictors of heart rate included air temperature (included as colder temperatures may cause naturally lower heart rates), hour (time of day of capture), prior capture behavior (Meancap—representative of the alertness of the fawn prior to capture), weight (proxy for age), year of capture (categorical to account for separate years of collection) and the sex of the fawn.

Latency was converted to a categorical binomial based on the median amount of seconds it took fawns to leave (dividing them into stayers ie >10 s and leavers ie <10 s). The predictors of latency included hour (time of day of capture), capture number, prior capture behavior (Meancap), sex of the fawn, visibility of the site, weight (proxy for age) and year of capture (categorical to account for separate years of collection). Time of day (hour) was included as fawns captured earlier in the morning may have recently been asleep or experiencing lower temperatures which could affect both heart rate and latency to leave. Capture number was included to account for fawns that were captured more than once during the fawn tagging period (and therefore take into account of potential capture habituation). Prior capture behavior (Meancap) relates to the alertness of the fawn prior to capture and was included to account for different behavioral responses as a result of the fawns state (eg a sleeping fawn would respond differently to an alert fawn who attempted to run, therefore with a starting higher physiological state). Controlling for prior behavior was included to ensure that observed differences in physiological and behavioral responses reflected trait variation rather than artifacts of the capture context. Sex was included as a categorical predictor to account for any differences between males and females. Visibility was included as an explanatory variable for latency as poorly concealed sites with lower levels of vegetative cover can result in more flighty behavior and lower latency times due to feelings of exposure and vulnerability. Weight was included (as a proxy for age) as older fawns are generally more alert and vagile than younger fawns. After data exploration and model comparison, some numerical predictors were included in either or both their singular and quadratic form to account for any nonlinearity in the data (Amin et al. 2021).

The predictors of begging were exactly the same as described in the previous model testing our first hypothesis. Random effects included an unstructured covariance matrix for individual identity (eartag), allowing estimation of variances and covariances among heart rate, latency, and begging behavior to capture consistent individual differences. A group ID random effect with trait-specific variances (no covariance) accounted for residual variation within group observations, capturing measurement error and unmodeled heterogeneity. This structure appropriately partitions variance across levels and traits for robust inference. Since begging behavior (to beg or not to beg) and latency at release (stayers vs leavers) are categorical variables, we constrained their prior variance of the fixed effects to 1 (Hadfield 2010), and we specified a weakly informative prior variance (V) and degrees of freedom (n) for random effects (*V* = 3, *n* = 0.002). Within-individual covariation between the begging behavior and neonatal personality traits was set to 0, since they were not measured at the same time (see Hadfield 2010). We ran the Monte Carlo Markov chains for 1,000,000 iterations again with a burn-in interval of 50,000 and a thinning interval of 500 in this case. A total of 1900 iterations were sampled to estimate parameters for this model.

For both models testing our a priori hypotheses, correlation coefficients at the among-individual level and repeatability estimates, along with their 95% credible intervals, were computed following Houslay and Wilson (2017). We visually inspected trace plots and checked that autocorrelation levels among samples of all variables were lower than 0.1. We checked the model convergence by using the Heidelberg stationarity test (Heidelberger and Welch 1983). To visualize the relationships between mothers' and fawns’ begging behavior, we extracted the posterior means of the random intercepts (BLUPs; Houslay and Wilson 2017) along with their uncertainties.

## Results

Data were collected across 382 observations of the herd during our feeding collection which included 255 neonate fawns born to 146 mothers across multiple fawning seasons (2018 to 2023, with begging of the fawns later on in life as well as of the other deer in the population collected from 2018 to 2024). We included fawns born from 2018 to 2023 (excluding 2024) in our first model looking at begging behavior as this meant all fawns were at least 1 year old during our last feeding observation period, giving them sufficient time to mature and engage in feeding interactions if they so wished. Across the dataset, 73 mothers had more than 1 offspring, with mothers producing an average of 1.76 offspring (SD = 0.94, range = 1 to 5).

**Figure 1:**
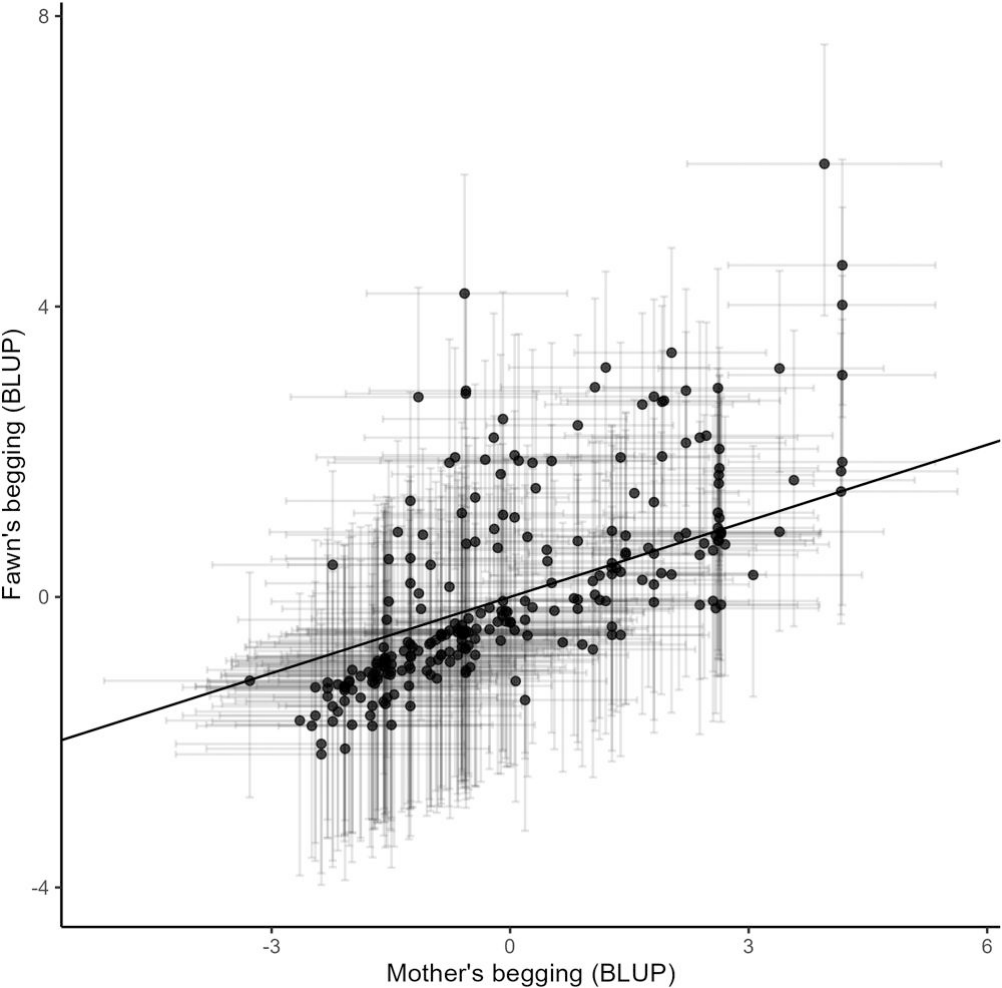
The relationship between the mother's begging behavior and the fawn's begging behavior as predicted by our univariate MCMCglmm. Each point represents the posterior mean of the estimated random intercept (BLUP) for a mother-offspring pair, derived from the MCMCglmm model. Horizontal and vertical bars show the standard errors of the BLUPs for mothers and fawns, respectively. The diagonal line indicates the expected slope based on the estimated heritability of begging behavior (*h*² = 0.35). Posterior means of the random intercepts (BLUPs) were used here for visualization purposes only.

We found that begging likelihood increased with herd size, number of people available to feed, age of the deer, in animals monitored for longer time, and that males tended to beg more (Fig. 1 and Table 1). Residual variance was fixed at 1, as specified by the model.

**Table 1:** Structure and output of the univariate model (MCMCglmm) used for the analysis of the among-individual correlation between mother's begging behavior and fawn's begging behavior. Posterior means with their associated 95% Credible Intervals and effective sample sizes of each of the explanatory variables included are given.

Fixed effects	Post. mean	95% CI lower	95% CI upper	Eff. samp	pMCMC
Intercept	−6.597	−7.658	−5.541	2504	<0.001***
Herd	−0.543	−0.869	−0.228	3225	<0.001***
Herd^2^	0.210	−0.038	0.464	3450	0.101
Sex (M)	0.527	−0.0723	1.142	3246	0.098
Month (July)	−0.073	−0.635	0.445	3208	0.792
Dow (Saturday)	0.047	−0.613	0.758	3450	0.898
Dow (Sunday)	0.406	−0.304	1.110	3166	0.263
√Monitoring time	0.365	−0.006	0.718	3450	0.048
√Monitoring time^2^	0.048	−0.193	0.337	3447	0.048*
√Total people	2.608	2.188	2.987	1602	<0.001***
√Total people^2^	−1.117	−1.391	−0.853	2048	<0.001***
Time of day	0.035	−0.267	0.318	3027	0.811
Time of day^2^	−0.011	−0.274	0.230	3116	0.956
Age	2.319	1.968	2.692	2359	<0.001***
Age^2^	−0.612	−0.783	−0.444	3260	<0.001***
Random Effects (Variance Components)					
Animal (G-Structure)	5.3	1.369	8.565	1768	
ID	2.112	0.091	5.109	2015	
Group ID	2.492	1.691	3.445	1650	
Year	0.886	0.154	2.124	3450	
Residual (units	1.00	1.00	1.00	0	
Heritability (*h*^2^)	0.350	0.123	0.538		

Significance codes indicate strength of evidence based on pMCMC values (*** < 0.001, ** < 0.01, * < 0.05), where smaller values indicate stronger support for an effect.

In regard to our main hypothesis, our first model allowed us to test for the heritability of behavior thanks to the inclusion of pedigree data (including mother—fawn pairings), and our heritability estimate using MCMCglmm was *h*^2^ = 0.35 (95% highest posterior density interval: 0.123 to 0.538, Fig. 1). Because the pedigree only includes maternal links, this estimate may partly capture shared maternal environmental effects and should be interpreted as an upper-bound on heritability. Begging behavior was moderately repeatable (*R* ≈ 0.49% CI: 0.403 to 0.57), demonstrating consistent differences among individuals across observations.

**Figure 2:**
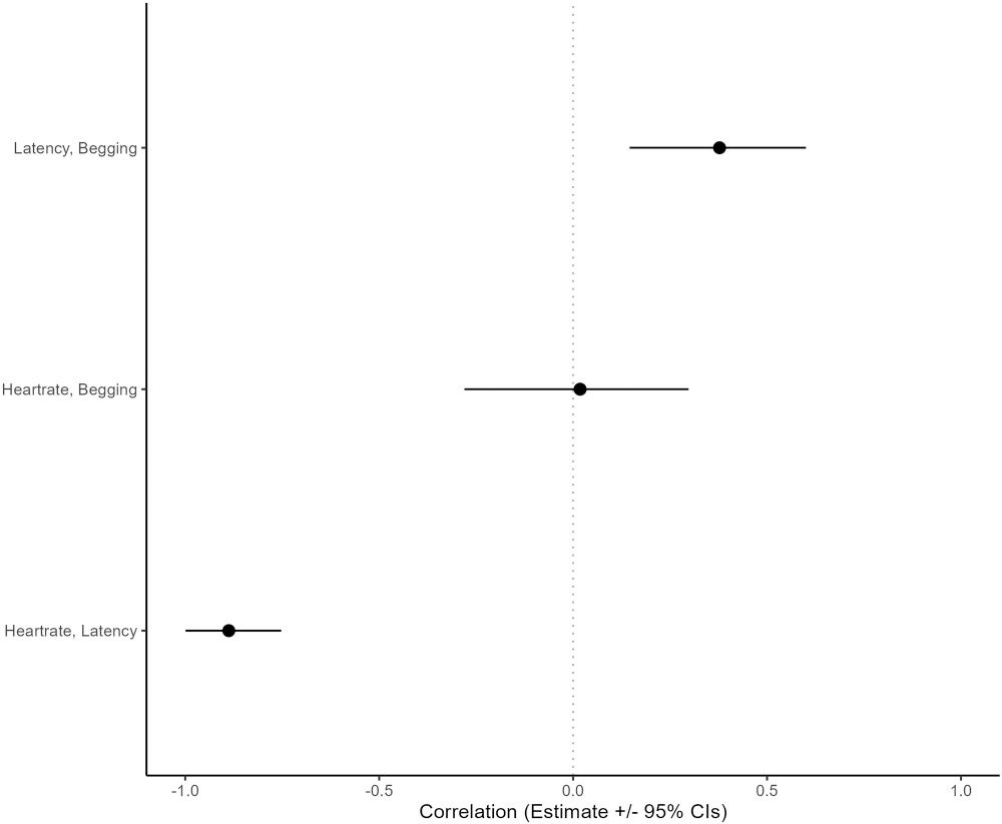
Among-individual correlations between heart rate at capture, latency to leave at capture and fawn’s begging behavior later in life from the multivariate model (MCMCglmm). Points represent posterior mean estimates of correlations, and horizontal lines indicate 95% credible intervals (CIs). Correlations whose credible intervals do not overlap zero indicate statistically meaningful effects.

The pedigree contains 376 individuals, including 160 siblings and 32 grandmother–grandchild pairs. There are no confirmed cousins because paternity is unknown, the study spans only a few generations, and females take time to mature and produce surviving offspring. High early fawn mortality and low survival among offspring of inexperienced mothers also limit the number of identifiable family links. In our second model linking neonatal personality to begging behavior (see Table S1 for full model parameter estimates), we found that the physiological (heart rate) and behavioral (latency to leave) neonatal personality traits were highly correlated (Fig. 2), with fawns coping better with human handling (lower heart rate) also less likely to leave soon after the release. Begging behavior was not related to heart rate; however it was clearly positively correlated with latency to leave as shown in Fig. 2. Our dataset included 554 fawns across 348 feeding observations (different to model 1 because these fawns did not need to be matched with mothers to be included). In relation to the effect of predictors explaining the response variables in our trivariate *MCMCglmm*, we found that the predictors of begging had similar patterns to those reported in the previous model (eg, males tend to beg more than females; total number of people available and age of the deer increased the likelihood of begging) and therefore has been included as Supplementary material (Table S1) to avoid redundancy. In terms of predictors of neonatal personality, heart rate was higher in heavier fawns (ie older, posterior mean = 0.48, 95% credible interval = 0.38 to 0.57), when ambient temperatures were higher (posterior mean = 0.28, 95% CI = 0.06 to 0.49), and in males when compared with females (posterior mean = 0.28, 95% CI = 0.15 to 0.42). Fawns were less likely to stay upon release if they were heavier (i.e older, posterior mean = −3.14, 95% CI = −4.19 to −2.19), males (posterior mean = −0.88, 95% CI = −1.70 to −0.11), and when released in highly visible sites (reduced understory vegetation and related protection—posterior mean = −0.10, 95% CI = −0.21 to −0.006). See Table S1 for the effect of all predictors included to explain the response variables (latency to leave, heart rate, and begging behavior).

## Discussion

Our aim was to test whether variation in begging behavior (ie, the tendency of wild animals to approach humans for food) shows intergenerational links that may be shaped, in part, by additive genetic effects, using maternal behavior in a peri-urban population of fallow deer as a focal point. Furthermore, we aimed to disentangle whether early-life personality traits contribute to the likelihood of begging later in life. We found a strong resemblance between mothers’ and fawns’ begging behavior, with fawns of mothers who beg more tending to beg themselves. The latent-scale heritability was estimated at 0.35; however, because our model does not explicitly separate maternal environmental effects from additive genetic variance, this estimate likely reflects a combination of both. Fully disentangling these sources of variation would require a cross-fostering design. In addition, we secondarily hypothesized that begging behavior was linked with neonatal personality, predicting that that individuals expressing risk-prone behavioral tendencies early in life would be more likely to approach humans later on. We have shown that bolder neonates (proxied by the tendency to stay upon release, ie the behavioral dimension of neonatal personality sensu Amin et al. 2021) tend to become beggars as they matured into adulthood, supporting our second hypothesis.

The observed resemblance in begging behavior among related individuals, particularly mothers and offspring, may therefore arise from both additive genetic effects and shared maternal environments. Because our model did not explicitly separate maternal effects from additive genetic variance, the heritability estimate should be interpreted cautiously as an upper-bound estimate of genetic contribution. As a result, any early-life conditions or maternal provisioning shared among offspring could inflate estimates of additive genetic variance. Nonetheless, the magnitude of the estimate (*h*^2^ = 0.35) indicates that a portion of the variation in begging behavior is associated with familial resemblance, though we cannot determine the relative contributions of genetic versus maternal environmental factors.

Interestingly, only the behavioral, but not the physiological, dimension of neonatal personality was linked to begging behavior later on in life. This is in accordance with previous research in our study system showing that—despite the 2 traits being highly correlated—they are linked to different life history traits (Amin et al. 2022a, 2022b). Behaviorally bolder fawns (those showing longer latencies to leave upon release) displayed reduced vigilance and more efficient foraging during their first-year (Amin et al. 2022a), whereas physiologically bolder fawns (characterized by lower heart rates during handling) had higher early-life survival (Amin et al. 2022b). Coupling these findings with our own, we see that fawns that express longer latencies, survive early life better and then grow up to express optimized foraging strategies, including increased begging behavior, leading to an overall fitness advantage over their conspecifics.

In addition to neonatal personality, our findings echo patterns previously noted by Griffin et al. (2022) that males have a higher tendency to beg than females. This may be due to male-biased risk taking behavior driven by greater energy requirements for reproductive investment (antler growth and rutting season in fallow deer, see Ciuti et al. 2004). Begging tendencies have also been shown to be more prevalent in older individuals (Griffin et al. 2022), so we hypothesized that older mothers would have been more likely to beg for food and so would their fawns.

A limitation of our study is that the pedigree includes only maternal links. As a result, the additive genetic variance estimated by our animal model may partially reflect maternal environmental effects, such as early-life provisioning or care. Therefore, our heritability estimates should be interpreted as an upper-bound, and some mother-offspring resemblance in begging behavior could be due to shared environment rather than genetics. Future studies incorporating paternal information or cross-fostering experiments could help disentangle these effects more precisely. Additionally, fallow deer show a U-shaped mortality curve meaning there is normally a high mortality rate for fawns with most of these deaths occurring soon after birth (Caughey 1966; Kjellander et al. 2012; Amin et al. 2022a). While our method of determining mother-fawn relationships is effective, it is limited to fawns that survive long enough to join the female herd (normally a few weeks). Creating complete genetic pedigrees would allow us to assign parentage to weaker fawns (who died a few days after birth) without relying on them surviving long enough to join the adult herd, therefore completing a complex puzzle we have started to build upon here with our research.

Begging behavior is a consistent, repeatable behavior that has emerged as a result of humans offering food to wildlife. Ever changing landscapes, driven by human activities like urbanization and agriculture, have meant that wildlife must become more adaptable to survive and reproduce (McKinney 2006; Ibanez-Alamo and Soler 2010). Selective pressures have led to many species adapting their behaviors to adjust to new environments (Seferta et al. 2001, Slabbekoorn and Peet 2003). Some species (and individuals within populations) exhibit greater behavioral plasticity than others in response to their surroundings, leading to more success during periods of human induced selective pressures. For example, many gull species have become synonymous with cities and exploit poor waste management for scavenging opportunities (Maciusik et al. 2010). Behavioral plasticity can afford wildlife both advantages and disadvantages in terms of health and reproductive success, which vary based on the context and foodstuffs offered during feeding interactions (Murray et al. 2016). Individuals willing to take riskier behavior, ie mothers willing to approach humans in our model deer population, are offered a selective advantage because they will produce heavier fawns at birth (Griffin et al. 2022), and humans reward their behavior with additional food, opening the door for artificial selection of beggars over their shyer conspecifics which has yet to be tested. Individuals that display less plasticity and are unable to adapt to high-disturbance environments are thought to be at a disadvantage and may be driven out over time and lost from urban populations altogether (Lowry et al. 2013), an hypothesis that requires testing in our model population and other ecological contexts where wildlife interact with humans.

These close contact interactions with wildlife are becoming increasingly popular across human-dominated landscapes and are known to also have consequences for humans as well. The vast majority of disease-causing pathogenic species infecting humans are zoonotic in nature (Taylor et al. 2001). We need only think of the epidemiological risk of zoonoses passing between domestic pets and humans, especially outdoor pets that regularly interact with wildlife, like domestic cats (*Felis catus*), to understand the risks involved (Salinas-ramos et al. 2021; Szentivanyi et al. 2024). Directly interacting with wildlife, and hand feeding in particular, coupled with low access to hygiene facilities in many public parks opens up a plethora of opportunities for disease transfer. Concurrent research in our study site has shown that fallow deer were seropositive for the Omicron variant of the COVID-19 virus (Purves et al. 2024). This leads to further concerns about the consequences of these interactions including the spread of zoonotic diseases and actions that must be taken by wildlife managers to control outbreaks, like vaccination and culling operations (Donnelly et al. 2006; Mathews 2009; Langton et al. 2022), which may have additional negative consequences for wildlife.

What influences willingness of wildlife to interact with humans in such close contact ways, and the passage of these tendencies needs to be better understood in order to predict the long term consequences of these activities. Many differences in behavior are known to have a genetic basis (Bolnick et al. 2003). Our results provide strong evidence for intergenerational resemblance in begging behavior but the mechanism of transmission, whether it is genetic inheritance or cultural transmission, remains to be fully understood.

Wildlife feeding and its associated behaviors are not limited to fallow deer and are commonly seen across most taxa from garden birds (Plummer et al. 2019), to terrestrial mammals (Knight 2010; Kojola and Heikkinen 2012; Usui and Funck 2018) and marine megafauna (Fitzpatrick et al. 2011; Senigaglia et al. 2020). It is important to note that there can be considerable side effects for the animals that do engage in these interactions. Mann et al. (2000) showed that wild dolphins that were regularly fed by humans had higher calf mortality, possibly due to human contact or pollution introduced into the environment through human presence and feeding activities. Provisioned diets are often not nutritionally sufficient in comparison to their natural diets as has been seen in Southern stingrays *(Dasyatis americana)*, fallow deer, and Australian magpies (*Gymnorhina tibicen*) and can cause health impacts in the long term (Ishigame et al. 2006; Semeniuk et al. 2007; Mclaughlin et al. 2022). The downsides associated with wildlife feeding begs the question should it be allowed at all (Newsone and Rodger 2008; Griffin and Ciuti 2023). The answer, we believe, lies in context and policy; unregulated feeding of random unsuitable food items can cause more harm than good (Griffin and Ciuti 2023). But sometimes wildlife feeding can be beneficial for conservation. For example, Starkey and delBarkco-Trillo (2019) documented how supplementary feeding supported native red squirrel (*Sciurus vulgaris*) populations that were being heavily outcompeted by invasive gray squirrels (*Sciurus carolinensis*) in the UK. Certainly, more research is needed to better understand the complex behavioral impacts of wildlife feeding and inform management policies that allow humans to enjoy and support a connection with nature without sacrificing an animal's health, promoting problematic behaviors, and negatively altering their community structures.

## Supplementary Material

arag034_Supplementary_Data

## Data Availability

Analyses reported in this article can be reproduced using the data provided by Faull et al. 2026.
