# Supplementary material for: Approaching humans for food is a behavior transmitted from mothers to offspring in a large herbivore
Source: Behav Ecol. 2026 Apr 6;37(4):arag034. doi: 10.1093/beheco/arag034 (PMC13231267; doi:10.1093/beheco/arag034)
Supplement: arag034_Supplementary_Data [file arag034_supplementary_data.docx]

**Supplementary materials:**


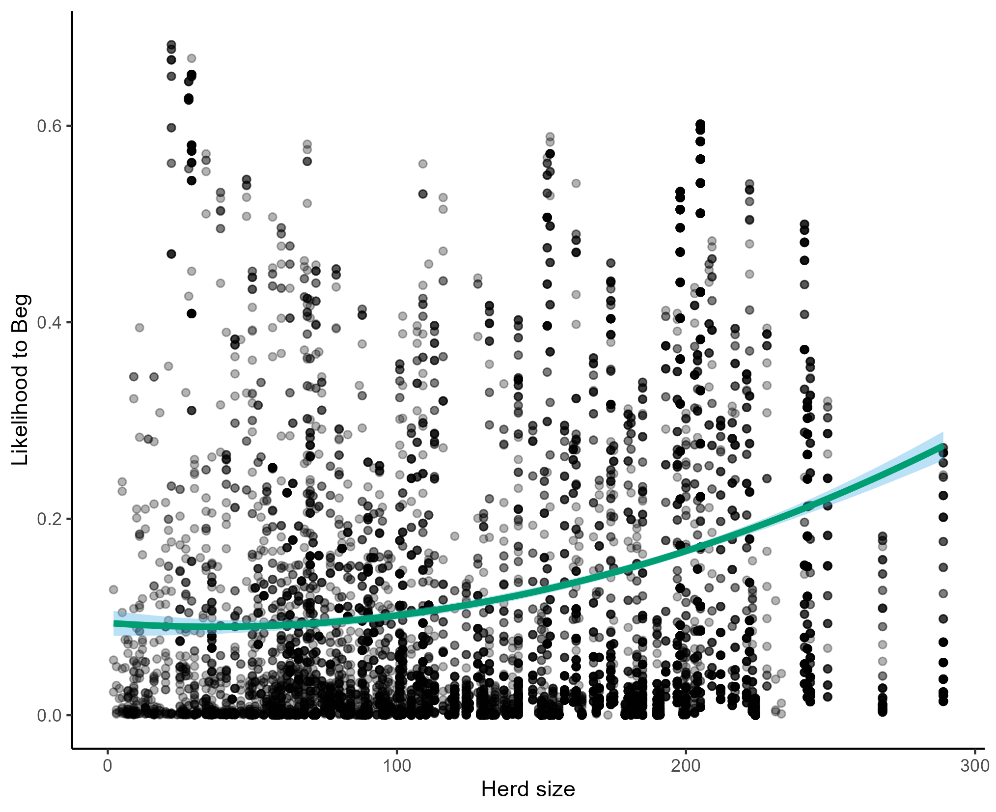


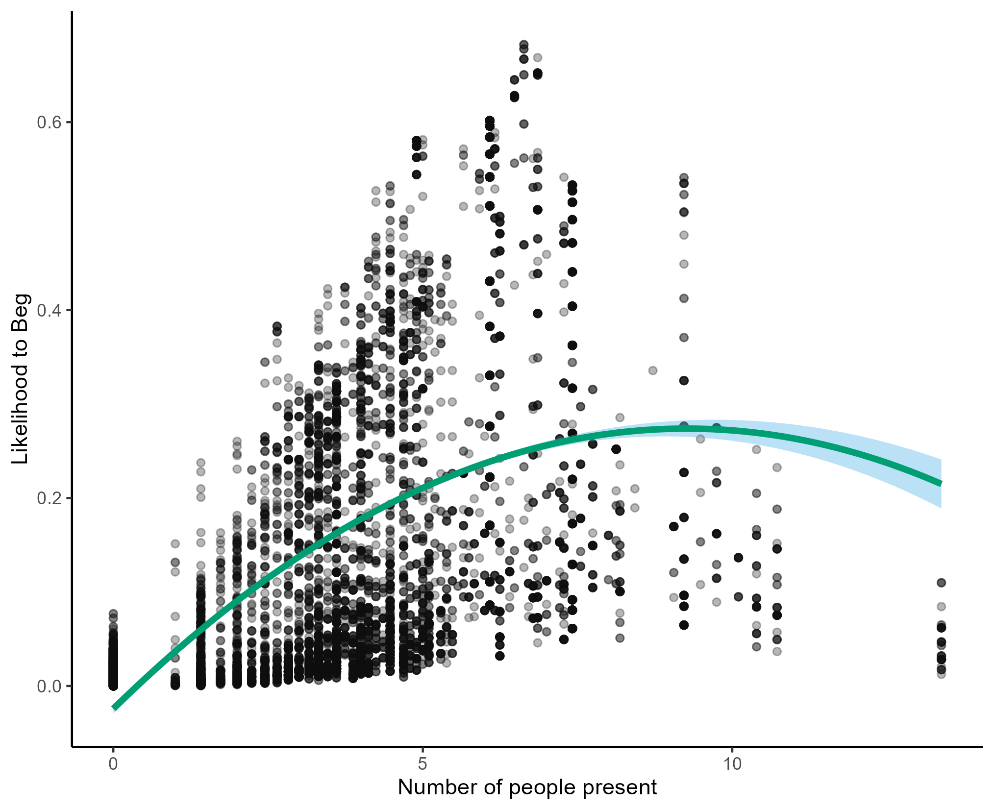


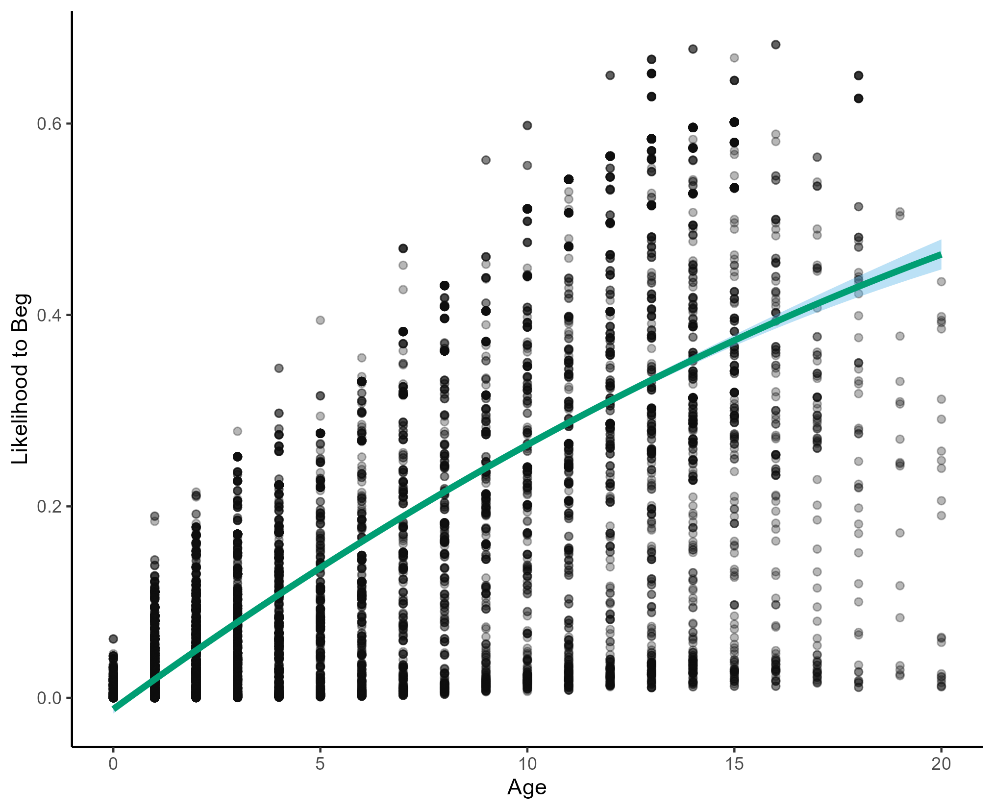


Figure S1: Effect of deer herd size (top plot), number of people present (middle plot), and age (bottom plot) on the likelihood of a fallow deer to beg (y‐axes) in the Phoenix Park, Dublin as predicted by MCMCglmm (model 1). Predicted effects are shown as lines surrounded by 95% confidence intervals along with row data displayed on the background.

Table S1: Structure and output of the multivariate model *(MCMCglmm* model 2 used for the analysis of the among-individual covariation between heart rate at capture, latency to leave at capture and begging behaviour. Posterior means and their associated 95% Credible Intervals (given in square brackets) of each of the explanatory variables included are given. Empty cells indicate that the explanatory variable was not included in the model for the respective response variables. Cells displayed in bold indicate statistically meaningful effects.

| Variable | Heart rate | Latency | Begging |
| --- | --- | --- | --- |
| Fixed effects | | | |
| Intercept | **-2.20 [-2.98,-1.34]** | **10.18 [4.18,14.11]** | **-9.06 [-11.19,-6.61]** |
| Air Temperature | **0.27 [0.05,0.49]** |  |  |
| Air Temperature^2^ | -0.03 [-0.07,0.01] |  |  |
| Hour |  | **-0.80 [-1.43, -0.21]** |  |
| Hour^2^ | **0.08 [0.01,0.14]** |  |  |
| Prior behaviour | 0.02 [-0.01,0.07] | **-0.31 [0.49, -0.14]** |  |
| Weight | **0.48 [0.39,0.58]** | **-3.14 [-4.19, -2.19]** |  |
| Year (2018) | **-0.25 [-0.48,-0.03]** | **-1.92 [-3.37, -0.61]** | 0.84 [-3.06, 4.92] |
| Year (2019) | -0.23 [-0.45, 0.04] | -0.21 [-1.54, 1.16] | 1.42 [-0.39, 3.36] |
| Year (2020) | -0.21 [-0.47, 0.03] | 0.72 [-0.55, 2.04] | 1.52 [-0.04, 3.03] |
| Year (2021) | **-0.26 [-0.49, -0.01]** | 0.59 [-0.69, 1.96] | 1.22 [-0.25, 2.69] |
| Year (2022) | 0.04 [-0.21, 0.27] | 0.69 [-0.58, 2.08] | -0.72 [-2.05, 0.49] |
| Year (2023) |  |  | -1.11 [-2.52, 0.14] |
| Sex (m) | **0.27 [0.15, 0.42]** | **-0.88 [-1.70, -0.11]** | **0.92 [0.40, 1.46]** |
| Capture (1) |  | 1.23 [-1.53, 4.88] |  |
| Capture (2) |  | 0.76 [-1.77, 4.53] |  |
| Capture (3) |  | -0.12 [-2.99, 3.54] |  |
| Visibility |  | **-0.10 [-0.21, -0.01]** |  |
| Herd Size |  |  | -0.37 [-0.85, 0.15] |
| Herd Size2 |  |  | -0.18 [-0.68, 0.29] |
| Month (June) |  |  | 0.48 [-0.35, 1.23] |
| DOW (Friday) |  |  | **-1.39 [-2.32, -0.35]** |
| DOW (Saturday) |  |  | -0.73 [-1.65, 0.07] |
| √Monitoring time |  |  | 0.35 [-0.41, 1.10] |
| √Monitoring time^2^ |  |  | 0.12 [-0.57, 0.82} |
| √Total people |  |  | **2.63 [2.04, 3.26]** |
| √Total people^2^ |  |  | **-1.33 [-1.76, -0.88]** |
| Average time |  |  | -0.84 [-5.11, 2.64] |
| Average time^2^ |  |  | 0.44 [-1.09, 2.47] |
| Age |  |  | **2.04 [1.58, 2.46]** |
| Age^2^ |  |  | **-0.38 [-0.58, -0.18]** |
| Random effects (G-Structure) | | | |
| Heartrate | 0.18 [0.09, 0.27] | 0.18 [0.09, 0.27] | 0.02 [-0.29, 0.33] |
| Latency | -1.02 [-1.45, -0.62] | 7.96 [3.46, 14.14] | 2.62 [0.82, 4.44] |
| Begging | 0.02 [-0.29, 0.33] | 2.62 [0.82, 4.44] | 6.37 [4.45, 8.29] |
| Random effects (R-Structure) | | | |
| Observation level | 6.30 [0.00, 23.49] | 1.93 [0.00, 12.11] | 3.83 [2.26, 5.50] |
